# Excess Epicardial Fat and Myocardial Remodeling After Mitral Valve Surgery

**DOI:** 10.3390/jcdd13080345

**Published:** 2026-07-23

**Authors:** Irina Lyapina, Elena Dren, Anastasia Kareeva, Aleksander Stasev, Eugenia Gorbatovskaya, Julia Yur’eva, Maria Khutornaya, Irina Mamchur, Olga Barbarash

**Affiliations:** Federal State Budgetary Institution “Research Institute for Complex Issues of Cardiovascular Diseases”, Boulevard of Academician L.S. Barbarash, 6, Kemerovo 650002, Russia

**Keywords:** valvular heart disease, mitral valve, excess epicardial fat, surgical correction, perioperative myocardial remodeling

## Abstract

**Objective**: This study aimed to assess the relationship between excess epicardial fat and the patterns of perioperative myocardial remodeling in patients undergoing surgical correction of mitral valve (MV) disease. **Methods**: A total of 148 patients with acquired non-infectious MV disease scheduled for surgical correction under cardiopulmonary bypass were screened in this prospective observational non-randomized study. Preoperative computed tomography (CT) of the heart was performed to assess epicardial adipose tissue (EAT) volume. Transthoracic echocardiography (Echo), including evaluation of left ventricular (LV) global longitudinal strain (GLS), right ventricular (RV) free-wall longitudinal strain, and RV systolic function (3D Echo), was conducted preoperatively, as well as postoperatively during one year after surgery. Analysis of postoperative myocardial remodeling and complications within one year after surgery was performed. Patients were divided into groups before surgical correction of MV based on the (1) EAT volume, associated with atrial fibrillation (AF) presence (EAT volume less than or > 115.1 cm^3^ by CT), and (2) EAT volume, associated with the presence of at least three metabolic factors (EAT volume less than or ≥100.6 cm^3^). **Results**: Prior to MV correction, Echo showed that patients with EAT volume > 115.1 cm^3^ exhibited larger left and right atrial (LA/RA) volumes and more pronounced RV systolic dysfunction. An EAT volume of >115.1 cm^3^ was associated with a 4.6-fold increase in the odds of detecting a preoperative TAPSE value < 1.7 cm (OR: 4.6 [95% CI: 1.2543; 16.7481]; *p* = 0.02). In the early postoperative period, patients with EAT volume > 115.1 cm^3^ exhibited larger RA dimensions and higher RV end-systolic volumes, as well as impaired RV–pulmonary artery coupling. At the one-year follow-up, patients with EAT volume > 115.1 cm^3^ exhibited larger indexed atrial volumes and basal RV dimensions. By the one-year follow-up, the group with EAT volume ≤ 115.1 cm^3^ was characterized by dynamic improvements, including a 10.7% increase in LV GLS (*p* = 0.02), a 33.6% reduction in the indexed LA volume (*p* = 0.004), a 28% reduction in the LV mass index (*p* = 0.003), and a 10.3% reduction in the LV end-diastolic dimension (*p* = 0.01). Furthermore, this group exhibited a 15% increase in LV stroke volume (*p* = 0.009), a 17.6% increase in TAPSE (*p* = 0.02), and a 6.5% increase in RV ejection fraction (*p* = 0.04) (3D Echo), none of which were observed in the group with EAT volume > 115.1 cm^3^. Patients with EAT volume ≥100.6 cm^3^ had more pronounced impairment of LV GLS before and one month after surgery compared with those with EAT < 100.6 cm^3^ (*p* = 0.046; *p* = 0.045). One month after surgery, worsening of RV GLS was observed specifically in the group with EAT ≥ 100.6 cm^3^ (*p* = 0.031). By the one-year follow-up, significant improvement in RV systolic function was observed only in the group with EAT volume < 100.6 cm^3^. **Conclusions**: The presence of excess epicardial fat (verified by cardiac CT) in cardiac surgery patients with acquired MV disease is associated with less favorable preoperative remodeling of both the left and right cardiac chambers and impaired reverse myocardial remodeling within one year post-surgery. Further studies in larger, independent cohorts are needed to confirm the prognostic and clinical relevance of the EAT cut-off in patients with mitral valve disease.

## 1. Introduction

The process of myocardial remodeling is dynamic and occurs at various levels, ranging from the cellular level (cardiomyocytes) to the organ level (alterations in cardiac geometry), involving changes in the size as well as the structural and functional characteristics of the heart chambers [1,2]. Myocardial-remodeling processes in patients with acquired valvular heart disease (VHD) clearly depend on the type and etiology of the defect, the severity of intracardiac and pulmonary circulation hemodynamics, and the nature and clinical significance of comorbid conditions [2]. Obesity is currently one of the most prevalent comorbid conditions among individuals with cardiovascular diseases. According to WHO data, the prevalence of obesity has doubled over the past twenty years, and as of 2022, the number of obese individuals worldwide has been approaching one billion [3].

A number of studies have demonstrated the contribution of obesity to the process of myocardial remodeling in individuals with cardiovascular diseases [4,5]. However, there is an «obesity paradox», demonstrating a lower risk of mortality following cardiac surgery in obese individuals compared with those without obesity [6]. This implies the limited clinical and diagnostic use of body mass index (BMI) as a measure of obesity.

It is important to note that it is the visceral adipose tissue (VAT) that serves as a specific predictor of pathological myocardial remodeling, whereas there is no established correlation between BMI values and the pattern of remodeling [7,8,9].

Researchers are currently showing particular interest in epicardial adipose tissue (EAT) as a component of visceral obesity due to its immediate proximity to the myocardium and their shared blood supply network.

To date, accumulated data regarding the influence of VAT, including EAT, on the structural and functional characteristics of the heart chambers have been obtained primarily from patients with coronary pathology and heart failure [10]. The influence of visceral obesity components on the cardiac valvular apparatus has been evaluated primarily in experimental studies [11,12]. This determines the relevance of further clinical studies aimed at investigating various components of obesity and their influence on the pattern of myocardial remodeling in patients with acquired VHD [13,14].

The objective of this study was to evaluate the association between excess epicardial fat and the pattern of perioperative myocardial remodeling in patients undergoing surgical correction of MV disease.

## 2. Materials and Methods

A total of 148 patients undergoing surgical correction of non-infectious mitral valve (MV) disease under cardiopulmonary bypass were screened for a prospective, observational, non-randomized, single-center study conducted between 2024 and 2025.

All patients provided informed consent to participate in this study. This study was conducted in accordance with the Good Clinical Practice standards and the principles of the Declaration of Helsinki. The study protocol was approved by the Institutional Review Board of the Research Institute for Complex Issues of Cardiovascular Diseases (Minutes No. 7, dated 16 September 2024).

Inclusion criteria: Age between 35 and 75 years; presence of acquired mitral valve disease caused by rheumatic heart disease, connective tissue dysplasia syndrome, or degenerative lesions; planned surgical correction of the mitral valve, either isolated or in combination with aortic or tricuspid valve repair, under cardiopulmonary bypass; and signed informed consent to participate in this study.

Exclusion criteria: Mitral valve disease etiology associated with infectious endocarditis; hemodynamically significant stenosis of coronary or peripheral arteries requiring revascularization; body weight exceeding 120 kg; tachyform of atrial fibrillation (AF) or atrial flutter (AFL) at the time of examination, preventing multispiral computed tomography (CT) imaging; presence of an implanted pacemaker; absolute contraindications for CT; severe respiratory failure (grades II–III); pregnancy or lactation; severe liver dysfunction (>9 points on the Child–Pugh scale, class C); requirement for hemodialysis, history of myocardial infarction, acute cerebrovascular accident, or pulmonary embolism within six months prior to hospitalization for surgical correction; severe neurological deficit; and psychiatric disorders.

The baseline characteristics of the patients prior to MV correction are presented in Table 1.

Isolated correction of MV was performed in 93 patients (62.84%) and double-valve correction in 55 patients (37.16%) (MV correction and tricuspid valve repair: *n* = 34; correction of combined mitral and aortic valve disease: *n* = 21). Intraoperative procedures included ligation of the left atrial (LA) appendage in 35.8% of patients, radiofrequency ablation of the pulmonary veins for AF in 35.8%, and the bi-atrial MAZE IV procedure in 6.76%.

Two-dimensional and three-dimensional transthoracic echocardiography (Echo) was performed prior to the surgery on days 7 and 30 and one year after MV correction. In addition to the standard echocardiographic protocol, myocardial strain and right ventricular (RV) systolic function were assessed using an expert-class system (Vivid E9, GE Healthcare, Chicago, IL, USA). Echo and evaluation of the Echo parameters for all patients were performed by the same doctor.

**Figure 1:**
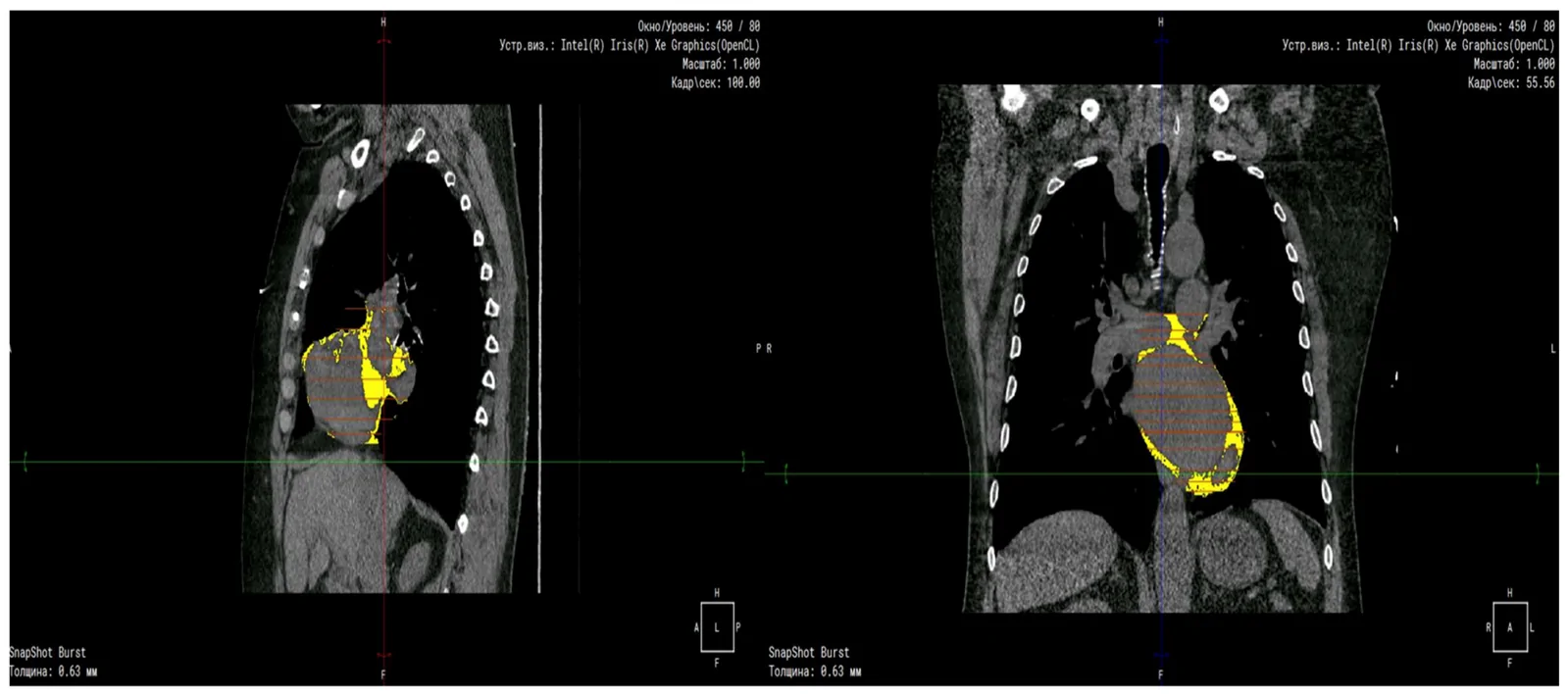
Assessment of epicardial adipose tissue volume using cardiac multislice computed tomography. Note: Epicardial adipose tissue is highlighted in yellow.

To identify excess epicardial fat and quantify EAT volume, patients underwent cardiac CT prior to surgical MV correction. Imaging was performed on a 64-slice scanner (Siemens Somatom, Munich, Germany) with synchronization of ECG recording (Figure 1). The attenuation thresholds used for EAT segmentation were from −50 to −160 Hounsfield units.

CT and evaluation of the CT parameters for all patients were performed by the same doctor.

Currently, there is no universally recognized criterion for epicardial obesity or excess epicardial fat [15]. Therefore, ROC analysis was required to identify the threshold for epicardial fat volume that determines the presence or absence of its excess. The presence of AF/AFL in patients with MV disease was selected as the factor for determining the EAT threshold via ROC analysis. The choice of AF/AFL as the rhythm disorder was based on the previously confirmed association between epicardial fat and the presence of AF/AFL in patients with cardiovascular diseases [16,17].

In our study, an EAT volume exceeding 115.1 cm^3^ (by the CT data) was associated with the presence of AF/AFL (AUC = 0.714; 95% CI: 0.57–0.83; *p* = 0.003), according to the ROC analysis, with a sensitivity of 52% and a specificity of 84.6%.

Among the included patients (n = 148), there was an equal distribution of females (47.3%) and males (52.7%) (median age: 65.0 [53.0; 67.8] years).

Based on the AF/AFL-derived EAT threshold, patients were divided into two groups: 62 patients with EAT volume > 115.1 cm^3^ and 86 patients with EAT volume ≤ 115.1 cm^3^, according to cardiac CT data.

It is well established that the commonly accepted critical threshold for visceral adipose tissue (area > 130 cm^2^ by CT) was derived based on its association with a high risk of metabolic complications. Given existing evidence that EAT volume correlates with markers of fibrosis and the inflammation characteristic of metabolic syndrome, we also performed ROC analysis to identify a critical EAT volume threshold by CT in a cohort of cardiac surgery patients with MV disease. Metabolic syndrome comprises five key components, and the diagnosis typically requires at least 3 of the following: waist circumference ≥ 80 cm in women and ≥94 cm in men; impaired carbohydrate metabolism (fasting plasma glucose ≥ 5.6 mmol/L, or previously diagnosed type 2 diabetes mellitus/impaired glucose tolerance); arterial hypertension/use of antihypertensive medications; hypertriglyceridemia (blood triglycerides ≥ 1.7 mmol/L); and low HDL-C (<1.0 mmol/L in men and <1.2 mmol/L in women) [18].

Thus, the results of the ROC analysis in the present study showed that an EAT volume of ≥100.6 cm^3^ was a statistically significant predictor of the presence of any three components of metabolic syndrome (high metabolic risk), with a sensitivity of 76.7% and specificity of 69.4% (AUC = 0.748; 95% CI: 0.631–0.864; *p* < 0.001). This EAT volume may be considered as a criterion for pathological epicardial fat deposition in our category of patients with acquired VHD (metabolic-derived EAT threshold). Patients were stratified into those with EAT ≥ 100.6 cm^3^ (*n* = 76) and EAT < 100.6 cm^3^ (*n* = 72).

**Figure 2:**
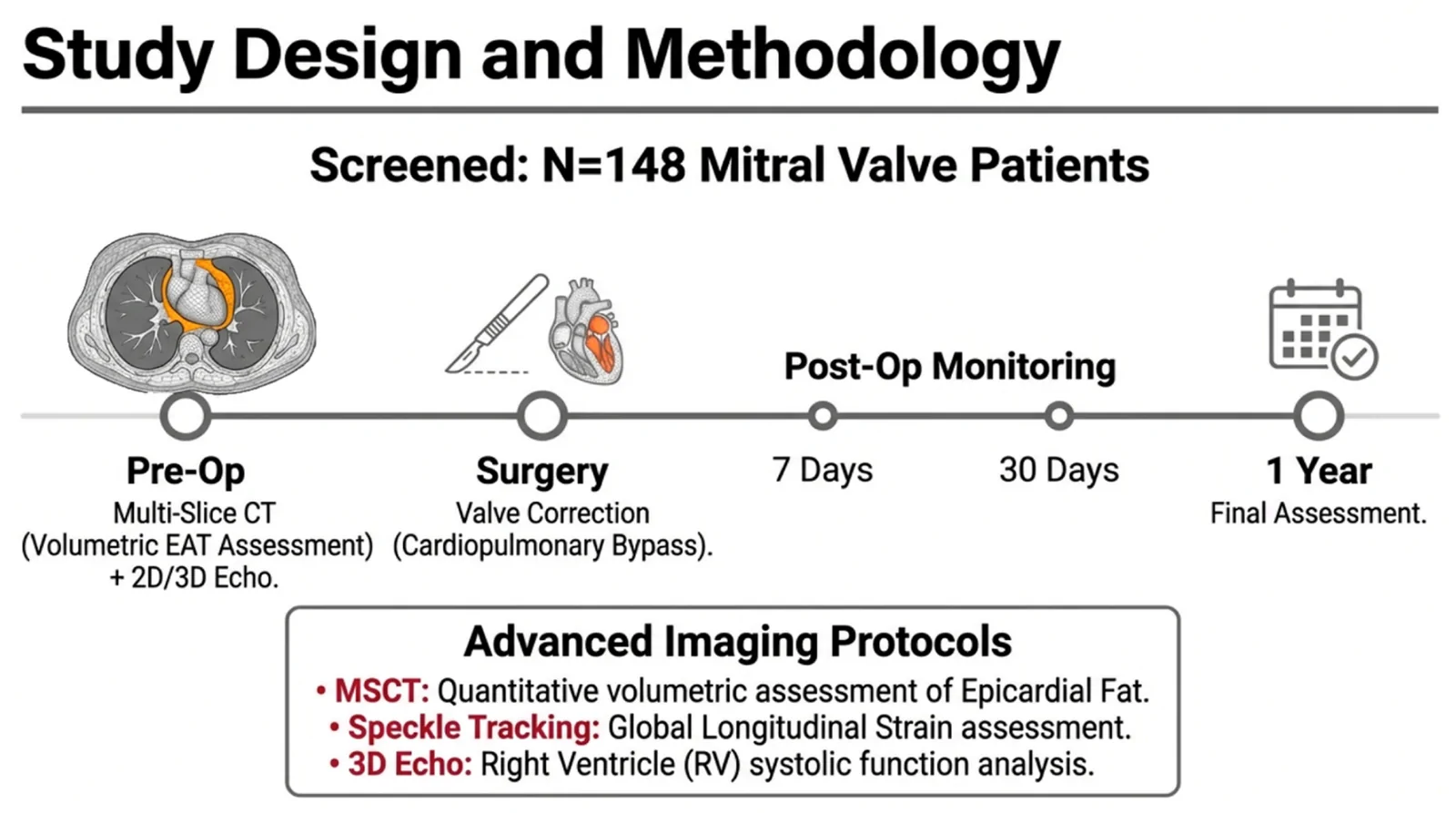
Study design.

The study design is presented in Figure 2.

Statistical data analysis was performed using standard non-parametric methods with the MedCalc software (version 20). Sample size calculation was performed using the Cochran Sample Size Calculator. Quantitative variables are presented as medians and interquartile ranges (Me [LQ; UQ]); qualitative variables are expressed as percentages. The Mann–Whitney U test was used to compare quantitative parameters between groups. Differences in qualitative variables were assessed using Pearson’s chi-squared test. Intragroup changes over time were evaluated using the Wilcoxon signed-rank test. Correction for multiple comparisons was performed using Dunn’s test with Holm’s correction and Pearson’s chi-square test with Holm’s correction. The results of logistic regression analysis are presented as odds ratios (ORs) and levels of statistical significance (*p*-values). The linear regression analysis results are reported as B-coefficients and *p*-values. Receiver operating characteristic (ROC) curves were used to calculate threshold values (*p* < 0.05; area under the curve (AUC) > 0.6). The critical level of statistical significance was set at *p* < 0.05.

## 3. Results

There were no significant differences between patients with and without excess epicardial fat regarding sex (31 females (50.0%) in the EAT volume > 115.1 cm^3^ group vs. 39 (45.3%) in the EAT volume ≤ 115.1 cm^3^ group; *p* = 0.35). The groups were also comparable in age (65.0 [61.75; 69.0] years for the group with higher EAT volume vs. 58.5 [51.0; 67.0] years for the group with lower EAT volume; *p* = 0.11).

Rheumatic etiology of MV disease was more common in patients with EAT volume > 115.1 cm^3^ (62.9% vs. 34.9%; *p* = 0.04). Connective tissue dysplasia predominated in the non-high EAT volume group (51.16% vs. 19.35%; *p* = 0.03).

Patients with EAT volume > 115.1 cm^3^ were twofold less likely to present with MV regurgitation (33.87% vs. 69.8%; *p* = 0.017), while combined MV disease was observed twice as frequently as in patients without excess EAT (41.9% vs. 16.1%; *p* = 0.005). Patients in the EAT volume > 115.1 cm^3^ group had a more severe functional status; notably, while no patients in the non-high EAT volume group had NYHA Class IV chronic heart failure (CHF), it was observed in 9.7% of those with higher EAT volumes (*p* = 0.037).

Patients with EAT volume > 115.1 cm^3^ had a higher prevalence of AF/AFL (71.0% vs. 38.4%; *p* = 0.006) and a higher incidence of high-grade ventricular extrasystole (Lown grades IVa–V) (27.4% vs. 9.3%; *p* = 0.04). Body mass index was higher in the EAT volume > 115.1 cm^3^ group (32.4 [28.0; 37.1] vs. 23.8 [21.98; 26.6] kg/m^2^; *p* < 0.001). No other differences in comorbidities were identified.

Preoperatively, patients received standard therapy according to guidelines for CHF [19]. Significant differences regarding EAT volume were found in the use of beta-blockers and anticoagulants, which were taken by significantly more patients with EAT volume > 115.1 cm^3^. This finding is likely attributed to the higher prevalence of high-grade ventricular extrasystole and AF/AFL in this group.

**Figure 3:**
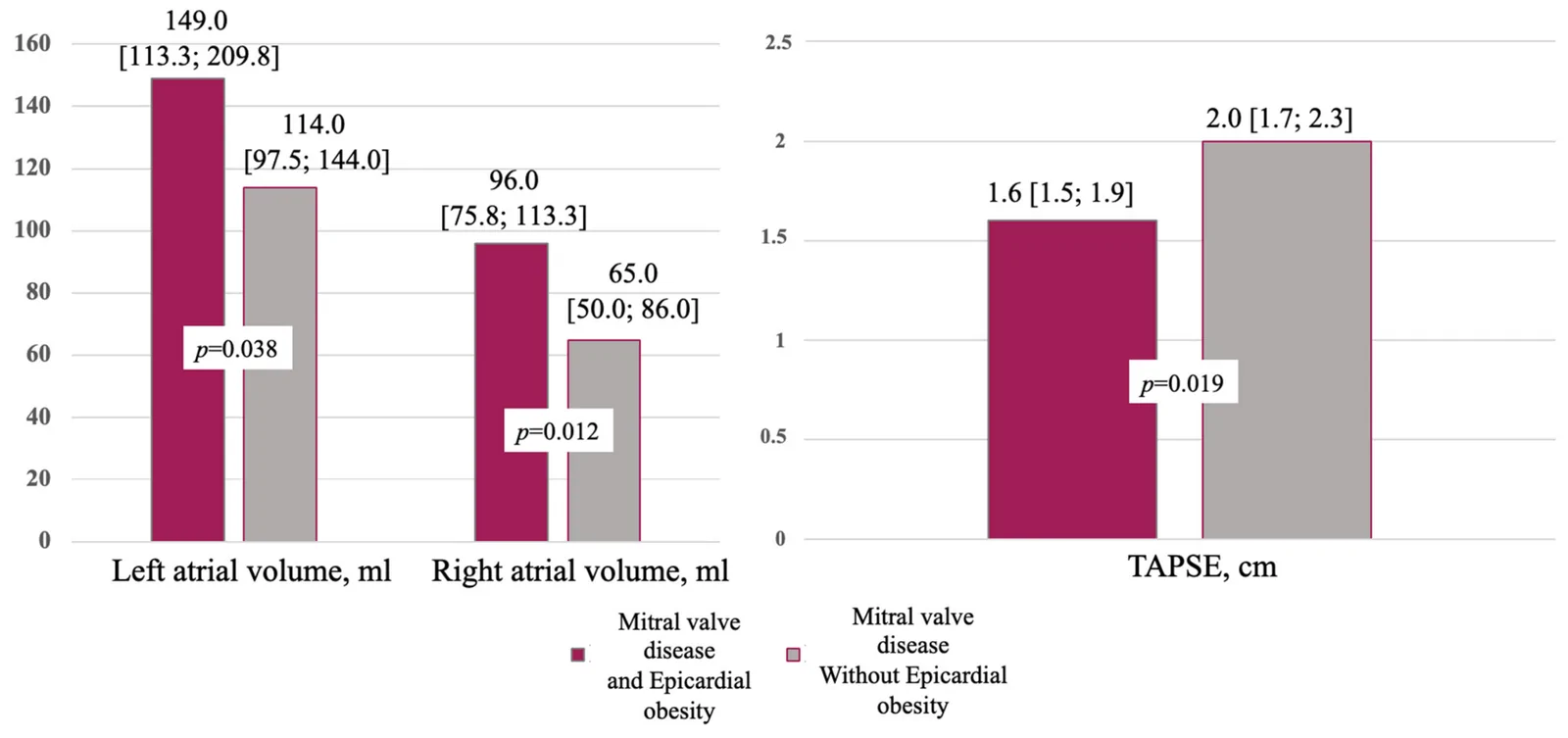
Differences in atrial volumes and right ventricular systolic function depending on the presence of excess epicardial fat in patients prior to MV correction. Notes: Data presented as Me [LQ;UQ]. The Mann–Whitney U test was used to compare quantitative parameters between groups.

The next stage involved assessing the association between EAT volume and the pattern of left and right heart chamber remodeling prior to MV correction. According to Echo results, patients with EAT volume > 115.1 cm^3^ were characterized by larger atrial volumes and a more pronounced decrease in RV systolic function (reduced tricuspid annular plane systolic excursion (TAPSE)) (Figure 3).

According to the results of univariate logistic regression analysis, an EAT volume exceeding 115.1 cm^3^ is associated with an increased risk of reduced RV systolic function (TAPSE < 1.7 cm) prior to MV correction (OR: 4.6 [95% CI: 1.2543–16.7481]; *p* = 0.021).

In the multivariate analysis conducted to assess the direct contribution of EAT to impaired RV function (TAPSE), the significance of an EAT volume > 115.1 cm^3^ as a predictor of RV dysfunction remained robust (Table 2), even after adjusting for other factors potentially affecting RV systolic function.

However, when AF was included in the analysis, the significance of EAT volume was eliminated, and the statistical significance of EAT volume was attenuated. The presence of preoperative AF/AFL in patients with MV disease is associated with a reduction in TAPSE, with a B-coefficient of −0.396 (*p* < 0.001).

According to the logistic regression analysis, an EAT volume exceeding 93.9 cm^3^ (representing the median value for the entire patient cohort) was associated with an increased likelihood of impaired left ventricular (LV) global longitudinal strain (GLS) (OR: 3.75; 95% CI: 1.076–13.073; *p* = 0.038).

When patients were divided into groups by preoperative EAT volume measured by cardiac CT, less favorable reverse remodeling was observed during the postoperative follow-up in the group with an EAT volume of > 115.1 cm^3^ compared with the lower-EAT-volume group (Table S1—Supplementary Material). One year after surgery, patients with EAT volume > 115.1 cm^3^ had a significantly larger LA diameter, indexed LA volume, and LA area compared with those with a lower EAT volume. During the postoperative period, LV volumes and LV mass were also significantly higher in patients with EAT volume > 115.1 cm^3^. In the early postoperative period, patients with EAT volume > 115.1 cm^3^ were characterized by more severely impaired LV GLS (Table S1—Supplementary Material).

Regression analysis revealed that a 1 cm^3^ increase in EAT volume was associated with an increase in right atrial (RA) volume, with a B-coefficient of 0.2759 (*p* = 0.005) at one week and a B-coefficient of 0.2251 (*p* = 0.0303) at one month postoperatively. Furthermore, EAT volume was associated with increased RV end-systolic and end-diastolic volumes, as measured by 3D Echo, with B-coefficients of 0.1635 (*p =* 0.016) and 0.2332 (*p* = 0.016), respectively, at one week after the surgery.

Furthermore, each 1 cm^3^ increase in EAT volume was associated with an increase in RA area, with a B-coefficient of 0.04221 (*p* = 0.0308), and an increase in RV basal diameter, with a B-coefficient of 0.00321 (*p* = 0.039), at the one-year follow-up after MV correction. One year postoperatively, the proportion of patients with Grade III tricuspid regurgitation was significantly higher in the EAT volume > 115.1 cm^3^ group (*p* = 0.0424) (Table S1—Supplementary Material).

Postoperative cardiac remodeling dynamics were evaluated in patients following MV correction, stratified by the presence of excess epicardial fat.

Compared with preoperative values, more pronounced reverse remodeling of the left heart chambers was observed one year after surgery in the group with a lower EAT volume (≤115.1 cm^3^):

− The left ventricular mass index (LVMI) significantly decreased by 28% (*p* = 0.003);
− The left ventricular end-diastolic diameter (LVEDD) decreased by 10.3% (*p* = 0.01);
− The LA diameter decreased by 9.4% (*p* = 0.002), and the left atrial volume index (LAVI) decreased by 33.6% (*p* = 0.004).

In contrast, these parameters showed no significant changes in patients with EAT volume > 115.1 cm^3^.

Patients without excess epicardial fat demonstrated an improvement in LV GLS of 12.2% (*p* = 0.004) between the one-month and one-year follow-ups, while no significant dynamics were observed in the EAT volume > 115.1 cm^3^ group.

The patients with EAT volume ≤ 115.1 cm^3^ were characterized by a 14% decrease in LV end-systolic volume (*p* = 0.04) one month after valve correction, whereas this parameter increased in the higher-EAT-volume group (*p* = 0.009). A 15% increase in LV stroke volume (*p* = 0.009) was noted in the lower-EAT-volume group from the one-month to one-year follow-up.

When evaluating postoperative remodeling dynamics of the right heart chambers, the group with an EAT volume of ≤115.1 cm^3^ showed an increase in RV stroke volume by 76% (*p* = 0.003), RV ejection fraction by 6.5% (*p* = 0.04), and RV fractional area change by 12.2% (*p* = 0.005) from one month to one year, while no significant dynamics were observed in the EAT volume > 115.1 cm^3^ group. In the lower-EAT-volume group, TAPSE increased from 1.4 [1.2; 1.72] cm to 1.7 [1.5; 1.9] cm (*p* = 0.002) between the 1-month and 1-year postoperative periods, whereas no significant increase was observed in the AF/AFL- derived EAT volume threshold > 115.1 cm^3^ group, which had lower baseline TAPSE values.

As indicated earlier, the proportion of patients with preoperative AF/AFL was higher in the group with EAT volume > 115.1 cm^3^. An important question naturally arises regarding the primary causes underlying the identified patterns of myocardial structural changes: the pronounced effects of an elevated level of EAT may also be associated with the presence of AF/AFL in these patients. Therefore, echocardiographic parameters were compared according to the presence or absence of AF/AFL in patients with EAT volume > 115.1 cm^3^ and EAT volume ≤ 115.1 cm^3^.

One week after surgery, myocardial remodeling parameters in patients with preoperative AF/AFL stratified by EAT volume (>115.1 cm^3^ vs. ≤115.1 cm^3^) also showed significant differences in LA diameter (5.3 [5.0; 5.8] vs. 4.6 [4.3; 4.8]; *p* = 0.004), as well as LA volume and area (128.5 [108.0; 190.0] vs. 84 [56.3; 104.0]; *p* = 0.010), RA area (27.2 [20.8; 33.9] vs. 19.7 [14.9; 24.4]; *p* = 0.040), and estimated systolic pulmonary artery pressure (33.8 [29.6; 37.9] vs. 28.4 [25.6; 31.2] mmHg; *p* = 0.041). In addition, among patients in sinus rhythm, a significantly larger LA volume on day 7 was also observed in those with EAT volume > 115.1 cm^3^ (109.0 [100.5; 117.5] vs. 80.0 [69.5; 83.0] mL; *p* = 0.043), and the LV end-systolic and end-diastolic volumes on day 7 after MV correction were also significantly greater in the EAT volume > 115.1 cm^3^ group (61.0 [44.0; 79.0] vs. 35.0 [26.0; 45.5] (*p* = 0.023) and 114.7 [81.8; 147.5] vs. 80.2 [66.9; 93.4] (*p* = 0.039), respectively).

One month after valve correction, among patients with preoperative AF/AFL, those with EAT volume > 115.1 cm^3^ again demonstrated larger LA diameters (5.4 [4.9; 6.0] vs. 4.5 [4.4; 4.7]; *p* = 0.013) and LA volumes (134.4 [102.0; 166.8] vs. 93.1 [65.0; 121.2]; *p* = 0.041). Among patients in sinus rhythm, those with preoperative EAT volume > 115.1 cm^3^ had greater indexed RA volumes (47.5 [38.0; 50.0] vs. 24.0 [19.8; 28.3]; *p* = 0.039).

One year after the surgery, among patients with preoperative AF/AFL, the group with preoperative EAT volume > 115.1 cm^3^ had a significantly larger LA diameter (6.1 [5.4; 6.2] vs. 4.9 [4.4; 5.0]; *p* = 0.003) and indexed LA volume (66.0 [57.5; 95.5] vs. 43.5 [39.0; 59.0]; *p* = 0.0379) as well as a larger LVEDD (5.7 [5.4; 6.1] vs. 5.2 [4.8; 5.6]; *p* = 0.042).

These findings suggest that not only AF but also EAT volume is a factor determining the dynamics of postoperative myocardial remodeling parameters.

The following section presents the main differences between patients with MV disease according to the metabolic-derived EAT threshold (volume ≥ 100.6 cm^3^).

Patients with EAT ≥ 100.6 cm^3^ had a longer duration of HF symptoms: 12.00 [10.00; 36.00] vs. 9.50 [6.00; 24.00] months before valve correction (*p* = 0.043). Preoperatively, they covered a significantly shorter distance on the 6 min walk test: 355.00 (99.6; 383.75) vs. 461.06 (93.00; 492.50) m (*p* = 0.02). A history of AF/AFL was more frequent in the higher-EAT group patients: 46 (60.5%) vs. 31 (43%) (*p* = 0.046). Patients with excess epicardial fat had greater myocardial mass before surgery (265.00 [189.5; 340.0] vs. 230.10 [170.0; 280.9] g; *p* = 0.040) and more pronounced impairment of LV GLS (−16.3 [−18.9; −12.4] vs. −19.2 [−21.8; −15.6]%) compared with those who had EAT volume < 100.6 cm ^3^ (*p* = 0.046).

One month after the intervention, patients with EAT ≥ 100.6 cm^3^ had a larger RA area (20.90 [17.15; 24.00] vs. 18.00 [17.00; 19.67] cm^2^; *p* = 0.047) and more impaired LV GLS (−12.60 [−8.47; −18.9] vs. −14.29 [−10.11; −21.9]%; *p* = 0.045) compared with patients with EAT < 100.6 cm^3^.

By one month after surgery, worsening of RV GLS was observed specifically in the group with EAT ≥ 100.6 cm^3^ (preoperatively: −19.6 [−23.55; −13.80] vs. one month after valve correction −16.50 [−18.45; −13.55]%; *p* = 0.031). In the group with EAT < 100.6 cm^3^, no significant change in RV GLS was found at one month after surgery (−19.00 [−25.50; −16.30]% before correction vs. −17.10 [−20.45; −15.55]% at one month; *p* = 0.09).

One year after the intervention, patients with excess EAT (≥100.6 cm^3^), as before surgery, had greater LV myocardial mass: 194.00 [176.00; 249.00] vs. 169.00 [148.00; 207.00] g (*p* = 0.043). When assessing within-group dynamics from one month to one year after surgery according to EAT volume, a significant improvement in RV systolic function was observed only in the group without excess EAT (EAT volume < 100.6 cm^3^). Thus, TAPSE increased from a median of 1.47 to 1.73 cm (*p* = 0.006), whereas in patients with EAT ≥ 100.6 cm^3^, the change in TAPSE was not significant (from 1.48 to 1.57 cm; *p* = 0.24). RV FAC significantly increased in the group without excess EAT from a median of 40.73% to 46.87% (*p* = 0.027), whereas in patients with EAT ≥ 100.6 cm^3^, the change in RV FAC was not significant (median: 39.4% to 41.4%; *p* = 0.672).

When analyzing the incidence of early postoperative complications, a higher frequency of stroke/transient ischemic attack was observed in the AF/AFL-derived EAT volume threshold group (n = 6; 9.7%) during the early postoperative period (*p* = 0.047). This finding may be associated with the significantly higher prevalence of AF/AFL in this group (*p* = 0.014).

One patient developed ventricular fibrillation, which was fatal (6 months after MV surgery). This patient had EAT volume > 115.1 cm^3^ before MV correction. The death of the second patient was not related to cardiovascular causes.

The incidence of readmissions for HF decompensation and cardiac arrhythmias was significantly higher in the EAT volume > 115.1 cm^3^ group, which may be attributed to a higher prevalence of pre-existing AF in this patient cohort (Table 3).

One year after MV correction, the incidence of arrhythmias (AF/AFL) was significantly higher in the group with EAT volume > 115.1 cm^3^ compared with the group with a lower EAT volume: 29 (48.33%) vs. eight (9.3%) patients, respectively (*p* = 0.003). Among patients discharged from the hospital with AF/AFL (*n* = 54), sinus rhythm restoration during the one-year follow-up period occurred in 12 (22.2%) patients in the EAT group and 19 (22.1%) patients in the non-EAT group (*p* = 0.1625). No significant differences were observed in the NYHA functional class between the EAT and non-EAT groups one year after surgery (Table 3).

While dividing patients by EAT volume associated with metabolic factors (<100.6 cm^3^ or ≥100.6 cm^3^), the incidence of readmissions for HF decompensation and cardiac arrhythmias was also significantly higher in the excess-epicardial-fat group (14.47% and 9.21%) compared with those with EAT < 100.6 cm^3^ (*p* = 0.02; *p* = 0.049).

## 4. Discussion

Obesity is a well-established driver of myocardial remodeling in patients with cardiovascular diseases, as evidenced by extensive fundamental and applied studies [4,8]. Pathological expansion of VAT and EAT leads to adipocyte hypertrophy and hyperplasia. This triggers an adipokine imbalance and a shift toward a pro-inflammatory profile, resulting in tissue damage, myocardial hypertrophy, and fibrosis [19,20,21]. Recent studies indicate that excessive visceral adipose tissue, including EAT, serves as a key predictor of pathological LV remodeling, whereas BMI often shows no correlation with remodeling patterns [7,8,9].

Our study focused specifically on EAT due to its anatomical proximity to the myocardium and its potential impact on adverse cardiac chamber remodeling. Adipokines and other bioactive substances secreted by EAT maintain energy homeostasis and provide mechanical protection for the myocardium. However, its pathological expansion is associated with adipokine dysregulation and the active production of pro-inflammatory interleukins, which may affect cardiomyocytes [4,22]. Research indicates that an increased EAT volume is associated with unfavorable outcomes in patients with heart failure with preserved LV ejection fraction (HFpEF) [22,23,24]. Currently, most publications studying structural and functional cardiac impairments in individuals with visceral obesity focus primarily on patients with coronary pathology and CHF [10,21,22,23,24].

In our study, myocardial remodeling was assessed using both standard transthoracic echocardiography (cardiac chamber dimensions and volumes) and an expanded protocol. The latter included speckle-tracking echocardiography to evaluate ventricular myocardial strain, as well as RV systolic function analysis based on 2D and 3D echocardiographic data. Schulz et al. [23] in their study demonstrated that an increased EAT volume was associated with impaired GLS of the LV and LA in patients with heart failure and diastolic dysfunction (HFpEF).

Several other studies have also demonstrated a link between EAT and impaired LV systolic and diastolic function, LV enlargement, LA fibrosis, and electrophysiological remodeling, the onset and severity of AF, and arterial stiffness parameters in patients with HF and ischemic heart disease [25,26,27,28,29]. For the cohort of patients with acquired VHD, available data are limited to associations between EAT thickness and the presence or severity of left-sided heart valve calcification [4], as well as the association between EAT thickness, measured by Echo, and the degree of LV remodeling in moderate to severe aortic stenosis [14,30]. Furthermore, in age-associated valvular diseases, EAT thickness is directly correlated with the severity of comorbid conditions, such as diabetes mellitus and obesity, as well as advanced patient age.

There is also evidence suggesting a correlation between EAT volume (measured by 3D echocardiography) and adverse cardiovascular and neurological events one year after transcatheter aortic valve implantation [31]. Another study demonstrated that patients undergoing transcatheter aortic valve intervention with a low EAT volume (<49 cm^3^ on CT) and high EAT attenuation (≥−86 HU) had higher all-cause mortality (log-rank *p* = 0.02 and *p* = 0.01, respectively). This association remained significant even after adjusting for age, sex, and clinical characteristics (HR: 1.71 (*p* = 0.02) and HR: 1.73 (*p* = 0.03), respectively) [32].

To date, no studies have investigated the relationship between epicardial adipose tissue thickness or volume and the patterns of myocardial remodeling in cardiac surgery patients with MV disease of various etiologies across a wide age range.

The conducted study is the first to demonstrate a significant association between EAT and structural–functional changes in both the left and right cardiac chambers in the cohort of surgical patients with MV disease. This relationship was observed both preoperatively and during the one-year follow-up period following surgical correction.

An important objective of our study was to identify an EAT threshold that would reflect adverse perioperative myocardial remodeling in cardiac surgery patients with MV disease. Many studies have previously demonstrated a relationship between EAT thickness/volume and the risk of developing AF. Using ROC analysis, in our study, we initially defined an EAT volume of > 115.1 cm^3^, which served as the AF/AFL-derived EAT threshold.

An EAT volume exceeding 93.9 cm^3^, as measured by CT, is associated with a more pronounced impairment of LV global longitudinal strain before surgery. Meanwhile, a higher EAT volume (exceeding 115.1 cm^3^) is associated with increased atrial volumes and deterioration of RV systolic function. Notably, the contribution of excess epicardial fat to the development of RV dysfunction remains significant even after adjusting for factors such as age, sex, type of VHD, comorbid profile, and a high probability of pulmonary hypertension based on Echo data.

Perioperative reverse myocardial remodeling serves as a reflection of the functional status, quality of life, and prognosis of surgical patients with acquired VHD. In the early postoperative period, patients with EAT volume > 115.1 cm^3^ exhibited larger RA dimensions and RV end-systolic volume, as well as impaired RV–pulmonary artery coupling. At the one-year follow-up, patients with EAT volume > 115.1 cm^3^ were characterized by larger dimensions of the right-sided cardiac chambers.

The association between EAT and AF is well established. Oba K. et al. evaluated the relationship between the EAT volume index and the prevalence of paroxysmal and persistent AF. Moreover, the association between the EAT volume index and persistent AF was modified by LA dimension, suggesting that atrial remodeling plays an important role in this relationship [33]. This study supports the relationship between EAT and AF, but it does not validate the use of AF/AFL as a state variable to define a general EAT threshold for “excess EAT or epicardial obesity”. A factor such as AF/AFL may independently contribute to the pattern of perioperative cardiac chamber remodeling and may act as an additional factor, alongside increased EAT volume, exacerbating this process.

Therefore, in our study, we graded the EAT volume based on the metabolic risk of patients with MV disease. Based on ROC analysis, we identified a metabolic-derived threshold for EAT volume of ≥100.6 cm^3^. Our study highlights that throughout the one-year postoperative period, EAT volume ≥ 100.6 cm^3^, in particular, exerts a more pronounced contribution to the adverse dynamics of myocardial remodeling. Intragroup dynamics analysis revealed that patients without excess EAT volume (<100.6 cm^3^) exhibited significant reverse myocardial remodeling across several parameters by the one-year follow-up, along with improvement in RV systolic function. In contrast, these favorable changes were not observed in the group with a higher EAT volume.

## 5. Conclusions

In this study, excess epicardial fat measured by cardiac CT was associated with less favorable preoperative remodeling of both the left and right cardiac chambers and with a less pronounced course of reverse myocardial remodeling during the one-year follow-up in patients undergoing surgical correction of acquired mitral valve disease. An internally derived EAT cut-off value of ≤ 115.1 cm^3^ identified a subgroup with more favorable postoperative LV reverse remodeling and better RV systolic function. However, this threshold should be considered study-specific, and further studies in larger, independent cohorts are needed to confirm the prognostic and clinical relevance of the EAT cut-off in patients with mitral valve disease.

### Study Limitations

A factor such as AF/AFL may independently contribute to the pattern of perioperative cardiac chamber remodeling and may act as an additional factor, alongside increased EAT volume, exacerbating this process. Therefore, the EAT volume threshold of >115.1 cm^3^ is AF-related and does not reflect excess epicardial fat across the entire population of patients with valvular heart disease. This threshold should be considered as an internally derived, AF/AFL-associated cut-off rather than a universally validated definition of epicardial obesity.

The cohort of included patients with valvular heart disease reflects real-world clinical practice in a tertiary valve center, where patients have different etiologies of mitral valve disease. Further studies are needed to clarify the influence of epicardial fat on myocardial remodeling in more homogeneous cohorts of patients with valvular heart disease (e.g., with a single etiology and isolated mitral or aortic valve correction).

## Figures and Tables

**Table 1:** Baseline clinical and medical history characteristics of patients with mitral valve disease prior to surgery.

Parameter	*n* = 148
Female, *n* (%)	70 (47.3)
Age, years, Me [LQ; UQ]	61.5 [51.3; 67.0]
Etiology of mitral valve disease, *n* (%)	
- Degenerative changes	16 (10.8)
- Rheumatic heart disease	69 (46.6)
- Connective tissue dysplasia syndrome	56 (37.8)
- Partial chordal rupture	7 (4.7)
Type of MV disease, *n* (%):	
- MV stenosis	21 (14.2)
- MV regurgitation	81 (54.7)
- Combined MV disease	46 (31.1)
Mixed valve disease, *n* (%):	
- With aortic valve stenosis/regurgitation	21 (14.8)
- With secondary tricuspid regurgitation	44 (29.7)
Heart rhythm characteristics, *n* (%):	
- Sinus rhythm	69 (46.6)
- Atrial fibrillation/atrial flutter:	79 (53.4)
○ Paroxysmal	16 (10.8)
○ Persistent	37 (25.0)
○ Permanent	26 (17.6)
- Ventricular extrasystole, Lown grade IVa–V	25 (16.9)
Obesity (BMI ≥ 30 kg/m^2^), *n* (%)	42 (28.4)
Stages of chronic heart failure, *n* (%):	
- Pre-stage	7 (4.7)
- Class 1	141 (95.3)
- Class 2	0
Chronic heart failure functional class	
(NYHA)	5 (3.4)
FC I	77 (52.1)
FC II	60 (40.5)
FC III	6 (4.1)
Six-minute walk test (6MWT) distance, m, Me [LQ; UQ]	296.3 [292.6; 387.2]
Diabetes mellitus/impaired glucose tolerance, *n* (%)	24 (16.2)
Arterial hypertension, *n* (%)	106 (71.6)

**Table 2:** Data from the multivariate regression analysis demonstrating the independent association between EAT volume and reduced tricuspid annular plane systolic excursion (TAPSE) prior to mitral valve correction.

	B	SE	*p*
Intercept	2.952	0.397	<0.001
Female	−0.100	0.129	0.446
Age	−0.012	0.007	0.098
Mitral stenosis	−0.300	0.308	0.336
Chronic obstructive pulmonary disease	0.116	0.189	0.544
Type 2 diabetes mellitus	−0.034	0.155	0.830
Arterial hypertension	0.107	0.145	0.485
Coronary artery disease	−0.086	0.147	0.562
Preoperative estimated pulmonary artery systolic pressure ≥ 38 mmHg	−0.098	0.198	0.624
EAT volume, cm^3^	−0.003	0.001	0.044

**Table 3:** Causes of readmission and functional status in patients during the one-year follow-up after MV correction, stratified by the presence of preoperative excess epicardial fat (EAT volume > 115.1 cm^3^) assessed by computed tomography.

Parameter	Patients with MV Disease		*p*
	EAT Volume > 115.1 cm^3^ (*n* = 62)	EAT Volume ≤ 115.1 cm^3^ (*n* = 86)	
Hospitalizations for CHF decompensation, *n* (%)	11 (17.74)	0 (0)	0.0152
Arrhythmia-related hospitalizations, *n* (%)	7 (11.3)	0 (0)	0.0499
MV mechanical prosthesis dysfunction and re-operation, *n* (%)	0 (0)	1 (1.6)	0.2568
Death, *n* (%)	2 (3.2)	0 (0)	0.1905
CHF functional class (NYHA), *n* (%)			
I	In 60 patients 32 (53.33)	56 (65.1)	0.3906
II	22 (36.66)	27 (31.4)	0.7761
III	3 (5)	3 (3.5)	0.7418
IV	3 (5)	0 (0)	0.1901

## Data Availability

The original contributions presented in this study are included in this article. Further inquiries can be directed to the corresponding author.

## Supplementary Materials

The following supporting information can be downloaded at https://www.mdpi.com/article/10.3390/jcdd13080345/s1: Table S1: Comparative analysis of transthoracic echocardiographic dynamics in patients with acquired mitral valve disease postoperatively, stratified by the presence of excess epicardial fat as assessed by CT (EAT volume > 115.1 cm^3^).

## Abbreviations

AF	Atrial fibrillation
AFL	Atrial flutter
CHF	Chronic heart failure
GLS	Global longitudinal strain
EAT	Epicardial adipose tissue
LA	Left atrium
LV	Left ventricle
MV	Mitral valve
RA	Right atrium
RV	Right ventricle
VAT	Visceral adipose tissue
VHD	Valvular heart disease
