# Supplementary material for: Excess Epicardial Fat and Myocardial Remodeling After Mitral Valve Surgery
Source: J Cardiovasc Dev Dis. 2026 Jul 23;13(8):345. doi: 10.3390/jcdd13080345 (PMC13513360; doi:10.3390/jcdd13080345)
Supplement: Supplementary file 1 [file jcdd-13-00345-s001.zip › jcdd-4356357-supplementary.pdf]

Table S1. Comparative analysis of transthoracic echocardiographic dynamics in patients with acquired mitral valve disease postoperatively, stratified by the presence of excess epicardial fat as assessed by CT (EAT volume > 115.1 cm^3).

Parameter Me [Q1;Q3]	Group								*p* between groups	*p* – dynamics in group 1	*p* – dynamics in group 2
	EAT volume > 115.1 cm^3 (n=62, 1 year follow-up in n=60)				EAT volume ≤ 115.1 cm^3 (n=86)						
	Preoperative	Day 7	24 days	1 year	Preoperative	Day 7	24 days	1 year			
	1	2	3	4	5	6	7	8			
Left heart chambers, Me [Q1;Q3]											
LV mass, g	295.0 [199.75; 344.3]	–	217.0 [177.5; 287]	240.0 [182; 304]	229.0 [175; 297]	–	175.0 [138.25; 204.75]	183.0 [153; 221]	$p_{1.5}$=0.16<br>$p_{3.7}$=0.03<br>$p_{4.8}$=0.04	$p_{1.3}$=0.06<br>$p_{1.4}$=0.08<br>$p_{3.4}$=0.97	$p_{5.7}$=0.006<br>$p_{5.8}$=0.009<br>$p_{7.8}$=0.69
LVMI, g/m^2)	140.0 [103; 166.5]	–	109.0 [95; 144.75]	116.0 [89.5; 145.25]	129.0 [108; 155]	–	92.0 [82; 123]	101.0 [86; 111]	$p_{1.5}$=0.84<br>$p_{3.7}$=0.18<br>$p_{4.8}$=0.13	$p_{1.3}$=0.12<br>$p_{1.4}$=0.10<br>$p_{3.4}$=0.78	$p_{5.7}$=0.003<br>$p_{5.8}$=0.003<br>$p_{7.8}$=0.48
LVESD, cm	4.0 [3.47; 4.5]	3.5 [3.2; 3.8]	3.7 [3.4; 4.22]	3.9 [3.1; 4.3]	3.8 [3.1; 4]	3.4 [3.07; 3.92]	3.2 [3; 3.6]	3.3 [3.0; 3.5]	$p_{1.5}$=0.09<br>$p_{2.6}$=0.52<br>$p_{3.7}$=0.007<br>$p_{4.8}$=0.04	$p_{1.2}$=0.009<br>$p_{1.3}$=0.62<br>$p_{1.4}$=0.07<br>$p_{2.3}$=0.02<br>$p_{2.4}$=0.39<br>$p_{3.4}$=0.37	$p_{5.6}$=0.39<br>$p_{5.7}$=0.03<br>$p_{5.8}$=0.11<br>$p_{6.7}$=0.06<br>$p_{6.8}$=0.12<br>$p_{7.8}$=0.43
LVEDD, cm	5.9 [5.47; 6.32]	5.2 [4.7; 5.75]	5.2 [4.9; 5.95]	5.6 [5; 6]	5.8 [4.9; 6.7]	5.0 [4.67; 5.4]	4.9 [4.6; 5.3]	5.2 [4.9; 5.4]	$p_{1.5}$=0.45<br>$p_{2.6}$=0.68<br>$p_{3.7}$=0.05	$p_{1.2}$=0.0006<br>$p_{1.3}$=0.01<br>$p_{1.4}$=0.06	$p_{5.6}$=0.0004<br>$p_{5.7}$=0.0003<br>$p_{5.8}$=0.01

									$p_{4.8}$=0.08	$p_{2.3}$=0.03<br>$p_{2.4}$=0.04<br>$p_{3.4}$=0.87	$p_{6.7}$=0.05<br>$p_{6.8}$=0.37<br>$p_{7.8}$=0.02
LVESV, ml	50.0 [41.75; 62.5]	54.0 [36.25; 62.25]	58.5 [39; 71.0]	55.5 [35.5; 70]	43.0 [34.5; 58]	36.5 [27.0; 47.0]	37.0 [32.75; 49]	37.0 [30; 51]	$p_{1.5}$=0.16<br>$p_{2.6}$=0.009<br>$p_{3.7}$=0.02<br>$p_{4.8}$=0.13	$p_{1.2}$=0.24<br>$p_{1.3}$=0.74<br>$p_{1.4}$=0.53<br>$p_{2.3}$=0.009<br>$p_{2.4}$=0.43<br>$p_{3.4}$=0.45	$p_{5.6}$=0.24<br>$p_{5.7}$=0.04<br>$p_{5.8}$=0.27<br>$p_{6.7}$=0.37<br>$p_{6.8}$=0.09<br>$p_{7.8}$=0.94
LVEDV, ml	142.0 [97.75; 167.5]	112.0 [84.75; 132.0]	126.5 [87; 142.5]	113.0 [89.5; 136]	126.0 [93.5; 153]	81.5 [69.0; 105.0]	99.0 [77.25; 118]	104.0 [89; 125]	$p_{1.5}$=0.39<br>$p_{2.6}$=0.02<br>$p_{3.7}$=0.05<br>$p_{4.8}$=0.61	$p_{1.2}$=0.02<br>$p_{1.3}$=0.07<br>$p_{1.4}$=0.05<br>$p_{2.3}$=0.04<br>$p_{2.4}$=0.91<br>$p_{3.4}$=0.29	$p_{5.6}$=0.0003<br>$p_{5.7}$=0.0002<br>$p_{5.8}$=0.12<br>$p_{6.7}$=0.005<br>$p_{6.8}$=0.0007<br>$p_{7.8}$=0.02
LVEF, %	61.0 [55.75; 64.5]	53.0 [47.5; 59.5]	54.0 [46.5; 59.5]	59.5 [45.5; 66]	64.0 [59.75; 66.5]	57.0 [46.0; 64.0]	60.0 [52; 62.5]	63.5 [56; 68]	$p_{1.5}$=0.10<br>$p_{2.6}$ =0.46<br>$p_{3.7}$=0.08<br>$p_{4.8}$=0.31	$p_{1.2}$=0.02<br>$p_{1.3}$=0.005<br>$p_{1.4}$=0.56<br>$p_{2.3}$=0.86<br>$p_{2.4}$=0.24<br>$p_{3.4}$=0.11	$p_{5.6}$=0.0007<br>$p_{5.7}$=0.04<br>$p_{5.8}$=0.30<br>$p_{6.7}$=0.007<br>$p_{6.8}$=0.03<br>$p_{7.8}$=0.05
LA, cm	5.8 [5.15; 6.65]	5.2 [4.8; 5.72]	5.2 [4.55; 5.65]	5.6 [4.95; 6.1]	5.2 [4.5; 5.6]	4.5 [4.2; 4.7]	4.45 [4.05; 4.7	4.7 [4.2; 5.0]	$p_{1.5}$=0.01<br>$p_{2.6}$ <0.0001<br>$p_{3.7}$=0.002	$p_{1.2}$=0.02<br>$p_{1.3}$=0.002<br>$p_{1.4}$=0.23	$p_{5.6}$=0.0002<br>$p_{5.7}$=0.0014<br>$p_{5.8}$=0.002

									$p_{4.8}$=0.0007	$p_{2.3}$=0.47<br>$p_{2.4}$=0.31<br>$p_{3.4}$=0.18	$p_{6.7}$=0.60<br>$p_{6.8}$=0.38<br>$p_{7.8}$=0.005
LA volume, ml	149.0<br>[113.25;<br>209.75]	125.0<br>[105.5;<br>176.5]	110<br>[94.25;<br>144.75]	137.5<br>[97.5;<br>174.5]	114.0 [97.5;<br>144]	81.0<br>[62.0;<br>89.75]	67.5<br>[59;<br>83]	75.0<br>[65; 84]	$p_{1.5}$=0.04<br>$p_{2.6}$ =0.0001<br>$p_{3.7}$=0.001<br>$p_{4.8}$=0.0012	$p_{1.2}$=0.13<br>$p_{1.3}$=0.0009<br>$p_{1.4}$=0.15<br>$p_{2.3}$=0.21<br>$p_{2.4}$=0.91<br>$p_{3.4}$=0.91	$p_{5.6}$=0.004<br>$p_{5.7}$=0.0001<br>$p_{5.8}$=0.005<br>$p_{6.7}$=0.001<br>$p_{6.8}$=0.75<br>$p_{7.8}$=0.28
LAind, ml/m2	74<br>[55;<br>93.5]	70.0<br>[52.5;<br>99.25]	56<br>[46.5;<br>61]	59<br>[45.75;<br>85]	61.0<br>[50;<br>75]	46.0<br>[35.0;<br>54.0]	35.5<br>[33; 44]	40.5<br>[34; 47]	$p_{1.5}$=0.16<br>$p_{2.6}$ =0.004<br>$p_{3.7}$=0.005<br>$p_{4.8}$=0.01	$p_{1.2}$=0.82<br>$p_{1.3}$=0.04<br>$p_{1.4}$=0.15<br>$p_{2.3}$=0.008<br>$p_{2.4}$=0.008<br>$p_{3.4}$=1.0	$p_{5.6}$=0.03<br>$p_{5.7}$=0.0004<br>$p_{5.8}$=0.004<br>$p_{6.7}$=0.03<br>$p_{6.8}$=0.53<br>$p_{7.8}$=0.45
LV GLS %	- 15.6<br>[- 18.9;<br>-13.37]	- 12.1<br>[-13.55;<br>-10.15]	- 12.35<br>[- 14;<br>-9.9]	-15.6<br>[-17.12;<br>-11.37]	- 19.0<br>[- 21.37;<br>- 13.25]	-14.3<br>[-16.4;<br>-11.6]	- 15.1<br>[- 16.2;<br>- 13.4]	-17.2<br>[-18.2;<br>-14.8]	$p_{1.5}$=0.09<br>$p_{2.6}$=0.04<br>$p_{3.7}$=0.008<br>$p_{4.8}$=0.10	$p_{1.2}$=0.20<br>$p_{1.3}$=0.22<br>$p_{1.4}$=1.0<br>$p_{2.3}$=0.67<br>$p_{2.4}$=0.14<br>$p_{3.4}$=0.56	$p_{5.6}$=0.0006<br>$p_{5.7}$=0.02<br>$p_{5.8}$=0.02<br>$p_{6.7}$=0.21<br>$p_{6.8}$=0.005<br>$p_{7.8}$=0.004
Right heart chambers, Me [Q1;Q3]											

RA volume, ml	96.0 [75.75; 113.25]	78.0 [68.0; 99.0]	73.0 [60; 95]	88.0 [62.5; 111.5]	65.0 [50; 86]	53.0 [42.5; 76.5]	50.0 [40.25; 60.25]	55.5 [50.5; 64]	$p_{1.5}$=0.01 $p_{2.6}$=0.006 $p_{3.7}$=0.003 $p_{4.8}$=0.02	$p_{1.2}$=0.04 $p_{1.3}$=0.04 $p_{1.4}$=0.02 $p_{2.3}$=0.62 $p_{2.4}$=0.31 $p_{3.4}$=0.84	$p_{5.6}$=0.20 $p_{5.7}$=0.007 $p_{5.8}$=0.08 $p_{6.7}$=0.74 $p_{6.8}$=0.92 $p_{7.8}$=0.32
RA area, cm^2	27.8 [24; 30.5]	23.7 [20.82; 32.55]	22.5 [19.9; 29.5]	25 [18.75; 28.85]	23.8 [16.9; 25.75]	19.0 [16.0; 23.1]	18 [16.45; 19.92]	19.35 [18; 22]	$p_{1.5}$=0.09 $p_{2.6}$=0.006 $p_{3.7}$=0.02 $p_{4.8}$=0.07	$p_{1.2}$=0.44 $p_{1.3}$=0.07 $p_{1.4}$= - $p_{2.3}$=0.81 $p_{2.4}$=0.63 $p_{3.4}$=0.31	$p_{5.6}$=0.15 $p_{5.7}$=0.16 $p_{5.8}$=0.46 $p_{6.7}$=0.87 $p_{6.8}$=0.60 $p_{7.8}$=0.68
RAind, ml/m2	49.0 [37.25; 61.5]	40.0 [32.0; 51.0]	39.0 [30.5; 48]	45.5 [32; 54.5]	36.5 [29; 44]	29.0 [23.0; 33.0]	26.5 [22.5; 34.5]	30.5 [27.5; 34.5]	$p_{1.5}$=0.05 $p_{2.6}$=0.03 $p_{3.7}$=0.02 $p_{4.8}$=0.04	$p_{1.2}$=0.04 $p_{1.3}$=0.01 $p_{1.4}$=0.02 $p_{2.3}$=0.93 $p_{2.4}$=0.51 $p_{3.4}$=0.73	$p_{5.6}$=0.36 $p_{5.7}$=0.07 $p_{5.8}$=0.07 $p_{6.7}$=0.41 $p_{6.8}$=0.65 $p_{7.8}$=0.38
Right ventricular basal diameter, cm	3.9 [3.37; 4.27]	4.2 [3.85; 4.35]	4.0 [3.67; 4.32]	4.2 [3.95; 4.4]	3.5 [3.17; 4]	3.9 [3.65; 4.0]	3.6 [3.2; 3.8]	3.8 [3.6; 4.2]	$p_{1.5}$=0.39 $p_{2.6}$=0.11 $p_{3.7}$=0.02 $p_{4.8}$=0.04	$p_{1.2}$=0.12 $p_{1.3}$=0.87 $p_{1.4}$=0.40 $p_{2.3}$=0.06 $p_{2.4}$=0.74 $p_{3.4}$=0.13	$p_{5.6}$=0.20 $p_{5.7}$=0.98 $p_{5.8}$=0.71 $p_{6.7}$=0.07 $p_{6.8}$=0.13 $p_{7.8}$=0.15

RV fractional area change, %	38.0 [30.5; 45]	34.0 [29.5; 41.0]	42.0 [36.75; 46.25]	41.5 [36.0; 47.0]	45.0 [36.8; 48.5]	39.0 [35.25; 44.25]	41.0 [38.5; 43.5]	46.0 [40.5; 48.0]	$p_{1.5}$=0.13<br>$p_{2.6}$=0.17<br>$p_{3.7}$=0.96<br>$p_{4.8}$=0.28	$p_{1.2}$=0.34<br>$p_{1.3}$=0.38<br>$p_{1.4}$=0.25<br>$p_{2.3}$=0.31<br>$p_{2.4}$=0.25<br>$p_{3.4}$=0.21	$p_{5.6}$=0.007<br>$p_{5.7}$=0.17<br>$p_{5.8}$=0.23<br>$p_{6.7}$=0.58<br>$p_{6.8}$=0.08<br>$p_{7.8}$=0.005
TAPSE, cm	1.6 [1.5; 1.9]	1.3 [1.1; 1.5]	1.5 [1.1; 1.65]	1.5 [1.2; 1.7]	2.0 [1.7; 2.3]	1.5 [1.3; 1.6]	1.4 [1.2; 1.72]	1.65 [1.5; 1.9]	$p_{1.5}$=0.02<br>$p_{2.6}$=0.16<br>$p_{3.7}$=0.86<br>$p_{4.8}$=0.12	$p_{1.2}$=0.02<br>$p_{1.3}$=0.04<br>$p_{1.4}$=0.23<br>$p_{2.3}$=0.21<br>$p_{2.4}$=0.17<br>$p_{3.4}$=0.73	$p_{5.6}$=0.0001<br>$p_{5.7}$=0.0002<br>$p_{5.8}$=0.009<br>$p_{6.7}$=0.004<br>$p_{6.8}$=0.004<br>$p_{7.8}$=0.002
ePASP, mmHg	50.0 [41.0; 61.25]	33.0 [25.75; 40.5]	33.0 [28.75; 38.5]	33.0 [26.5; 42.5]	42.0 [31.0; 60.0]	28.0 [24.0; 31.0]	28.0 [22; 30.0]	29.0 [24; 34.5]	$p_{1.5}$=0.45<br>$p_{2.6}$=0.13<br>$p_{3.7}$=0.047<br>$p_{4.8}$=0.43	$p_{1.2}$=0.0005<br>$p_{1.3}$=0.001<br>$p_{1.4}$=0.008<br>$p_{2.3}$=0.75<br>$p_{2.4}$=0.96<br>$p_{3.4}$=0.48	$p_{5.6}$<0.0001<br>$p_{5.7}$=0.0001<br>$p_{5.8}$=0.0008<br>$p_{6.7}$=0.44<br>$p_{6.8}$=0.06<br>$p_{7.8}$=0.19
RV-PA coupling, mm/mmHg	0.32 [0.26; 0.49]	0.37 [0.33; 0.46]	0.37 [0.35; 0.52]	0.39 [0.32; 0.60]	0.42 [0.3; 0.63]	0.5 [0.42; 0.65]	0.52 [0.4; 0.65]	0.55 [0.41; 0.71]	$p_{1.5}$=0.18<br>$p_{2.6}$=0.02<br>$p_{3.7}$=0.09<br>$p_{4.8}$=0.24	$p_{1.2}$=0.02<br>$p_{1.3}$=0.19<br>$p_{1.4}$=0.02<br>$p_{2.3}$=0.31<br>$p_{2.4}$=0.63<br>$p_{3.4}$=0.58	$p_{5.6}$=1.0<br>$p_{5.7}$=0.67<br>$p_{5.8}$=0.22<br>$p_{6.7}$=0.47<br>$p_{6.8}$=0.19<br>$p_{7.8}$=0.26

RV free wall global longitudinal strain, %	- 17.0 [- 23.3; - 13.47]	-12.55 [- 15.4; - 10.6]	- 15.55 [- 16.8; - 13.65]	- 15.1 [-19.17; - 13.92]	- 18.55 [- 24.3; -16]	-16.9 [- 18.8; - 14.0]	- 17.25 [- 19.9; - 16.5]	17.9 [-22.22; - 15.47]	$p_{1.5}$=0.29<br>$p_{2.6}$=0.15<br>$p_{3.7}$=0.08<br>$p_{4.8}$=0.09	$p_{1.2}$=0.80<br>$p_{1.3}$=0.69<br>$p_{1.4}$=0.33<br>$p_{2.3}$=0.30<br>$p_{2.4}$=0.43<br>$p_{3.4}$=0.56	$p_{5.6}$=0.0006<br>$p_{5.7}$=0.09<br>$p_{5.8}$=0.12<br>$p_{6.7}$=0.14<br>$p_{6.8}$=0.70<br>$p_{7.8}$=0.71
RVEDV, ml 3D Echo	116.0 [60.5; 121.25]	129.0 [113.0; 138.75]	128 [87.25; 135]	136.0 [118.0; 167.0]	96.5 [74; 122.5]	96.0 [81.5; 109.75]	86.5 [75; 117]	129.5 [112.0; 153.0]	$p_{1.5}$=0.87<br>$p_{2.6}$=0.03<br>$p_{3.7}$=0.04<br>$p_{4.8}$=0.77	$p_{1.2}$= -<br>$p_{1.3}$= -<br>$p_{1.4}$=0.31<br>$p_{2.3}$=0.68<br>$p_{2.4}$=1.0<br>$p_{3.4}$=0.63	$p_{5.6}$=0.11<br>$p_{5.7}$=0.28<br>$p_{5.8}$=0.13<br>$p_{6.7}$=0.10<br>$p_{6.8}$=0.01<br>$p_{7.8}$=0.01
RVESV, ml 3D Echo	59.0 [36; 67.5]	76.0 [56.75; 83.25]	70.0 [41.75; 83]	73.0 [61.0; 100.0]	52.5 [41.5; 64.5]	53.5 [43.0; 87.0]	47.5 [35; 69]	67.0 [53.0; 87.0]	$p_{1.5}$=0.85<br>$p_{2.6}$=0.04<br>$p_{3.7}$=0.19<br>$p_{4.8}$=0.46	$p_{1.2}$= -<br>$p_{1.3}$= -<br>$p_{1.4}$= 0.44<br>$p_{2.3}$=0.13<br>$p_{2.4}$=0.38<br>$p_{3.4}$= -	$p_{5.6}$=0.39<br>$p_{5.7}$=0.21<br>$p_{5.8}$=0.23<br>$p_{6.7}$=0.28<br>$p_{6.8}$=0.08<br>$p_{7.8}$=0.06
RVSV, ml 3D Echo	50.0 [31.75; 55]	48.0 [42.25; 59.5]	55.0 [44.25; 56.75]	59.0 [51.0; 76.0]	42.0 [37.5; 62]	46.0 [36.25; 50.75]	37.5 [34; 48]	66.0 [59.25; 77.75]	$p_{1.5}$=0.55<br>$p_{2.6}$=0.45<br>$p_{3.7}$=0.04<br>$p_{4.8}$=0.43	$p_{1.2}$= -<br>$p_{1.3}$= -<br>$p_{1.4}$=0.06<br>$p_{2.3}$=0.13<br>$p_{2.4}$=0.25<br>$p_{3.4}$=1.0	$p_{5.6}$=0.15<br>$p_{5.7}$=0.33<br>$p_{5.8}$=0.10<br>$p_{6.7}$=0.06<br>$p_{6.8}$=0.004<br>$p_{7.8}$=0.003

RVEF, %<br>3D Echo	46.0 [37; 47.75]	38.0 [35.25; 40.5]	45.0 [35.75; 45.75]	47.0 [42; 50]	48.5 [43; 54]	45.0 [38.5; 48.75]	46.0 [42; 49]	49.0 [44; 53]	$p_{1.5}$=0.09<br>$p_{2.6}$=0.12<br>$p_{3.7}$=0.25<br>$p_{4.8}$=0.27	$p_{1.2}$= -<br>$p_{1.3}$= -<br>$p_{1.4}$=0.50<br>$p_{2.3}$=0.81<br>$p_{2.4}$=0.13<br>$p_{3.4}$=0.12	$p_{5.6}$= 0.005<br>$p_{5.7}$=0.05<br>$p_{5.8}$=0.61<br>$p_{6.7}$=0.91<br>$p_{6.8}$=0.04<br>$p_{7.8}$=0.04
Severity of tricuspid insufficiency, n (%)											
Grade I	18 (29.0)	29 (46.8)	21 (33.9)	33 (55.0)	46 (53.5)	60 (69.76)	58 (67.44)	49 (57.0)	$p_{1.5}$=0.01<br>$p_{2.6}$=0.16<br>$p_{3.7}$=0.04<br>$p_{4.8}$=1.0	–	–
Grade II	25 (40.32)	11 (17.74)	11 (17.74)	3 (5)	10 (11.6)	14 (16.3)	5 (5.8)	10 (11.6)	$p_{1.5}$=0.01<br>$p_{2.6}$=0.75<br>$p_{3.7}$=0.15<br>$p_{4.8}$=0.13	–	–
Grade III	7 (11.3)	4 (6.45)	3 (4.4)	5 (8.33)	16 (18.6)	0 (0)	0 (0)	0 (0)	$p_{1.5}$=0.61<br>$p_{2.6}$=0.15<br>$p_{4.8}$=0.04	–	–
Grade IV	4 (6.45)	0 (0)	0 (0)	0 (0.0)	3 (3.5)	0 (0)	0 (0)	0 (0)	$p_{1.5}$=0.48<br>$p_{4.8}$=0.82	–	–

Note: GLS – global longitudinal strain; PAP – pulmonary artery pressure; PA – pulmonary artery; LV – left ventricle; LA – left atrium; EDV – end-diastolic volume; ESV – end-systolic volume; EDD – end-diastolic diameter; ESD – end-systolic diameter; MV – mitral valve; RV – right ventricle; RA – right atrium; PASP – pulmonary artery systolic pressure; TV – tricuspid valve; EF – ejection fraction; and TTE/Echo – transthoracic echocardiography.
